# Reliability and validity of Arabic translation of Medication Adherence Report Scale (MARS) and Beliefs about Medication Questionnaire (BMQ)–specific for use in children and their parents

**DOI:** 10.1371/journal.pone.0171863

**Published:** 2017-02-13

**Authors:** Mervat Alsous, Fadwa Alhalaiqa, Rana Abu Farha, Mariam Abdel Jalil, James McElnay, Robert Horne

**Affiliations:** 1 Department of Clinical Pharmacy and Therapeutics, Faculty of Pharmacy, Applied Science Private University, Amman, Jordan; 2 Faculty of nursing, Philadelphia University, Amman, Jordan; 3 Department of Biopharmaceutics and Clinical Pharmacy, Faculty of Pharmacy, The University of Jordan, Amman, Jordan; 4 Clinical and Practice Research Group, School of Pharmacy, Queen’s University Belfast, Belfast, United Kingdom; 5 Centre for Behavioural Medicine, UCL School of Pharmacy, University College London, London, United Kingdom; University of Nottingham, UNITED KINGDOM

## Abstract

**Objectives:**

to evaluate the reliability and discriminant validity of Arabic translation of the Medication Adherence Report Scale (MARS) and the Beliefs about Medication Questionnaire-specific (BMQ-specific).

**Methods:**

Having developed Arabic translations of the study instruments, a cross-sectional study was carried out between March and October 2015 in two multidisciplinary governmental hospitals in Jordan. An expert panel monitored the forward and backward translation of the MARS and BMQ. Standard Arabic was used (with no specific dialect inclusion) to allow greater generalisability across Arabic speaking countries. Once the Arabic translations of the questionnaires were developed they were tested for consistency, validity and reliability on a group of children with chronic diseases and their parents.

**Results:**

A total of 258 parents and 208 children were included in the study. The median age of participated children and parents was 15 years and 42 years respectively. Principle component analysis of all questionnaires indicated that all had good construct validity as they clearly measured one construct. The questionnaires were deemed reliable based on the results of Cronbach alpha coefficient. Furthermore, reliability of the questionnaires was demonstrated by test-retest intraclass correlation coefficients (ICC) which ranged from good to excellent for all scales (ICC>0.706). The Pearson correlation coefficient ranged from 0.546–0.805 for the entire sample which indicated a significant moderate to strong positive correlation between MARS and BMQ items at time 1 and 2. Reported adherence was greater than 59% using MARS-children and MARS-parents scales, and was correlated with beliefs in necessity and independent of the concerns regarding medications.

**Conclusion:**

The Arabic translations of both BMQ and MARS for use in children and their parents have good internal consistency and proved to be valid and reliable tools that can be used by researchers in clinical practice to measure adherence and beliefs about medications in Arabic speaking patient populations.

## 1. Introduction

Prevalence of chronic diseases among pediatric patients is increasing together with associated health care costs [1]. Chronic conditions have long-term health care consequences that can affect a child’s emotional, physical, and social development [2, 3]. Unlike adults, children with chronic disease face unique challenges such as treatment adherence, lifestyle modification, disease acceptance and eventually shifting to adult health care settings [2].

The burden of chronic diseases such as hypertension and diabetes is increasing in Jordan [4]. Therefore, its management and prevention are considered significant issues for health care providers [5]. In 2005, 50% of deaths in Jordan were considered due to chronic diseases [6]. By 2030, non-communicable diseases will be responsible for 66.7% of deaths in the Middle East region [7].

Adherence to medications is generally poor and is particularly challenging in chronic conditions [8]. A World Health Organisation report stated that adherence to chronic therapies in developed countries is approximately 50% with lower rates reported in developing countries [9]. On the other hand, the nonadherence rate in the Middle East is estimated to be as high as 88% [10]. Non-adherence to medication has several negative consequences such as repeated clinic visits, extending course of illness, poorly controlled symptoms and increased cost due to unnecessary hospital admissions [11]. Adherence in a chronic paediatric condition is a difficult situation to deal with on many levels as it does not only affect the child, but can have an impact on the whole family [12]. Quite often it is the parent who is responsible for the control and administration of medications and ensuring that the child's illness is managed appropriately. As both parent and child are involved, adherence is commonly reported from both parties.

The guidelines developed by the World Health Organization [9] and National Institute for Clinical Excellence (NICE) recommend that health care professionals explore patient’s beliefs, concerns and attitudes toward medication as these are considered the most important drivers of non-adherence among chronic disease patients [13–16].

Several studies have shown that patients who have concerns and negative attitudes towards their medications are less adherent than those who have positive beliefs and attitudes [5, 13, 16–18]. Thus, assessing and addressing factors that influence adherence such as patient attitudes and beliefs may improve regimen adherence and reduce the burden of chronic diseases [9].

Many instruments have been developed to explore non-adherence and patient's beliefs about medications such as the Medication Adherence Report Scale (MARS) developed by Horne. It has good validity and reliability [19–21] and the Beliefs about Medicines Questionnaire (BMQ) developed by Horne et al. (1999) [13]. The use of these self-report instruments to assess adherence and factors affecting adherence is cheap and straight forward [22], however, the lack of validated Arabic language questionnaires is a significant obstacle that prevents using such instruments in Arabic speaking populations.

Alhalaiqa et al (2015) found that the Arabic translation of the BMQ-12 (general) is valid and reliable to be used among Jordanian citizens and they recommended translation of other instruments for use in Arabic speaking regions [23]. The aim of the present research was to develop and assess the validity and reliability of an Arabic translation of the Medication Adherence Report Scale and Beliefs about Medication Questionnaire (specific) in order that they can be used in future studies to assess adherence in Jordanian patients. So the aim of this study was to evaluate the reliability and discriminant validity of an Arabic translation of the MARS and BMQ-specific questionnaires for use in children with chronic diseases and their parents using a patient sample that covers most areas in Jordan.

## 2. Materials and methods

### 2.1 Study design

A cross-sectional study was carried out in two multidisciplinary governmental hospitals in two different geographical locations of Jordan.

### 2.2 Translation

Translation of the MARS and BMQ specific into Arabic was carried out after obtaining permission from the developer of the original English translation [13]. Forward and backward translations of the questionnaires were carried out by two bilingual individuals with medical backgrounds (pharmacists). They independently translated the original English measures into Arabic, and then combined the translations in to a single Arabic translation. A further two bilingual translators then carried out backward translation into English. A final English translation of each measure was sent to the original author (Professor-Robert Horne) to obtain his authorisation and comments. No changes were suggested to the back-translated questionnaire and the authors were therefore satisfied with the Arabic translations. The final Arabic translations were tested on 42 candidates as a pilot trial to test for clarity and comprehensiveness of the Questionnaires. The results of this latter pilot study revealed that both questionnaires were clear and understandable to participants.

### 2.3 Ethical considerations

The study was conducted following the ethical standards outlined in the World Medical Association Declaration of Helsinki guidelines [24]. Ethical approval from the Jordanian Ministry of Health Scientific Research Ethics Committee was obtained on 28th Jan 2015 (Reference number: MB/1169) to carry out further testing in parents and children with chronic illness. Research governance permissions for the study were obtained in March 2015 from Al-Bashir and Al-Karak Governmental Hospitals to conduct the study. Written informed consent obtained from parents/guardians and assent from their children prior to their participation in the study. Participant's confidentiality was preserved throughout.

### 2.4 Participants and recruitment procedure

A convenience sampling method was employed at outpatient clinics at both study site hospitals over period of 8 months depending on logistics and throughput rate of the clinics. This type of sampling helped gain the participation of as many children with chronic disease (and their parents) as possible. The inclusion criteria were; child diagnosed with chronic disease; aged between 11 and 18 years; able to read and write Arabic; taking regular prescriptions, had no apparent cognitive deficit, and had a follow-up appointment at an outpatient clinic within the study sites.

Research assistants (pharmacists and nurses) were trained to administer the questionnaires in a consistent manner (to decrease the risk of assessment bias). During the study period, 258 parents and 208 children were recruited. Participating patients and parents were asked to fill both questionnaires for a second time, 2–4 weeks after their recruitment. Demographic data, i.e. medical and medication history, current medications and parental education and income were also collected using customised data collection forms.

### 2.5 Data collection instruments

Arabic translations of the MARS and the BMQ-specific were used to collect the data; the questionnaires are available from authors. The original MARS, developed by Horne, measures the frequency of non-adherent behaviours and it has two translations relating to the period covered, i.e. either general overview of adherence and specific adherence over the last month; the latter was used in the present study. The questionnaire consists of six questions relating to non-adherent behaviours and have to rate the frequency that is applicable to them for each of the items. Thus translations of the questionnaires are available i.e. (i) a parent/guardian translation (for completion by the child's carer) and (ii) a child translation (for completion by the child over 11 years old). For each translation, scores for each of the six items are summed and divided by 6 to give a scale score ranging from 1 to 5. Higher scores indicate higher levels of self-reported adherence. The child/patient questionnaire translation is the same as the parent/guardian questionnaire, except for the last item that is not relevant to the child/patient, i.e. “I don’t give it because my child refuses it”. Therefore scores for each of the five items are summed and divided by 5 to give a scale score ranging from 1 to 5 for each child. Higher scores indicate higher levels of self-reported adherence.

The BMQ (specific) is a validated instrument used to quantify patient or parent beliefs about specific medication for a particular illness. It does this by measuring the perceived Necessity of treatment compared to the Concerns about treatment held by the individual. High Concern scores and low Necessity scores have been correlated with high levels of non-adherence in a number of chronic illnesses [25].

There are 11 questions in the questionnaire, each is coded as either a Necessity belief (n) or a Concern (c) and participants must answer by showing how much they agree or disagree with each statement. There are 5 necessity questions and 6 concern questions, and the participant is not made aware of the coding. Each answer is scored between 1 (strongly disagree) and 5 (strongly agree). The total necessity score (divided by 5) and concern score (divided by 6) are calculated separately to give a scale score ranging from 1 to 5 before being compared to see if the participant’s overall view of their medications is that of necessity or concern. As for the MARS, separate questionnaires were administered to children and their parents/guardians. The items for the BMQ-specific administered to the participating children and completed by them were the same as the BMQ-specific administered to the parents/guardians with the exception that the term “My child’s ….” was replaced with “My….” within each question

### 2.6 Data analysis

Data were analysed using the statistical Package for Social Science (SPSS) version 20. The descriptive analysis was carried out using mean and SD for quantitative variables. The questionnaire’s internal consistency and reliability was assessed by measuring Cronbach’s alpha coefficient (α) for each dimension, values ≥ 0.70 were considered satisfactory [26]. Intraclass Correlation Coefficient (ICC) and Pearson Product Moment Correlation were used to test–retest reliability of the questionnaires. ICC test correlations were considered as good to excellent when r ≥0.75, good when r ranged between 0.5 and 0.7, as fair when r ranged between 0.25 and 0.50, and as little or no relationship when r was less than 0.25 [27]. The validity of the MARS and BMQ-specific for both parent and child translations was tested using exploratory Principal Component Analysis (PCA).

## 3. Results

### 3.1 Demographic characteristics

A total of 258 parents and 208 children were included in the study between March and October 2015. The median age of participated children was approximately 15 years and their age was between 11 and 18 years. A total of 58.5% of the children were female and 90.7% of them were medically insured. Parents were between 19 and 69 years of age and most of them were outside the medical field (95%). The majority of parents (62%) had intermediate monthly income between 420 and 630$ and 56.4% of parents had completed secondary school education. Participant demographic characteristics are summarised in Table 1.

**Table 1:** Demographic characteristics of the participants.

	Parent characteristics	Child characteristics
**Gender**		
Male	51 (20.2%)	86 (41.5%)
Female	201 (79.8%)	121 (58.5%)
**Age**		
Median (range)	42 years (19–69)	15 years (11–18)
**Medical insurance**		
Insured	228 (89.8%)	188 (90.8%)
Not insured	26 (10.2%)	19 (9.2%)
**Parent education**		
Not educated	29 (11.6%)	
School level (Primary)	19 (7.6%)	
School level (Secondary)	141 (56.4%)	
Primary degree	22.4 (22.4%)	
Postgraduate degree	2 (2%)	
**Parent occupation**		
Medical field	12 (5%)	
Other	227 (95%)	
**Parent income level**		
• >840$	1 (0.4%)	
• 630–840$	21 (8.7%)	
• 420–630$	150 (62%)	
• <420$	70 (28.9%)	
**Child disease**		
Endocrine		67 (32.4%)
Respiratory		56 (27.1%)
Genetic		29 (14%)
Neurology		18 (8.7%)
Oncology		17 (8.2%)
Nephrology		8 (3.9%)
Autoimmune disease		11 (5.3%)
Cardiology		1 (0.5%)
**Duration of the disease**		
• Median (range)		4 years (0.41–18)

### 3.2 Validity of questionnaires

#### 3.2.1 Principal component analysis for MARS and BMQ-specific questionnaires

Exploratory principal component analysis (PCA) was conducted on the Arabic translations of MARS and BMQ-specific scales in order to investigate construct validity for use by parents and children with chronic diseases. Essentially PCA was used to establish whether the items in each questionnaire were measuring one component i.e. non-adherence in the MARS questionnaires and necessities or concerns in the BMQ-specific questionnaires.

To determine whether the sample was large enough to perform principal component analysis the Kaiser-Meyer-Olkin (KMO) measure and Bartlett’s sphericity were calculated. The KMO is required to be above 0.5 for sampling adequacy [28] while a significant p value for the Bartlett’s test of sphericity indicates that the variables tested are correlated. The number of components being assessed by the questionnaire was established by examining Eigenvalues and a scree plot.

Eigenvalues describe how much variance is explained by each item in the questionnaire. The number of components with a value of 1 (Kaiser’s criterion; [28]) or above can indicate the number of components present, Additionally, the number of components above the point of sharp decline in Eigenvalues on a scree plot can also be used to identify the number of components principally explaining the variation in the data.

**Fig 1:**
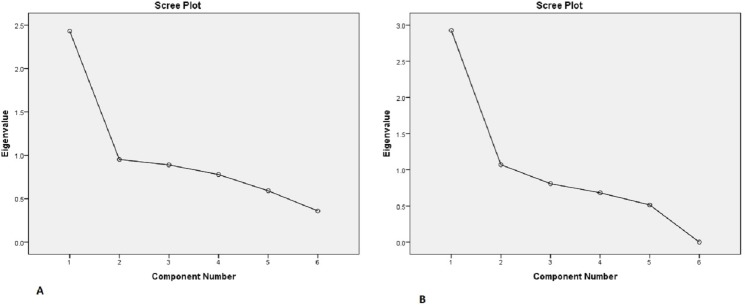
Scree plot of parental MARS (A) and children MARS (B).

**Fig 2:**
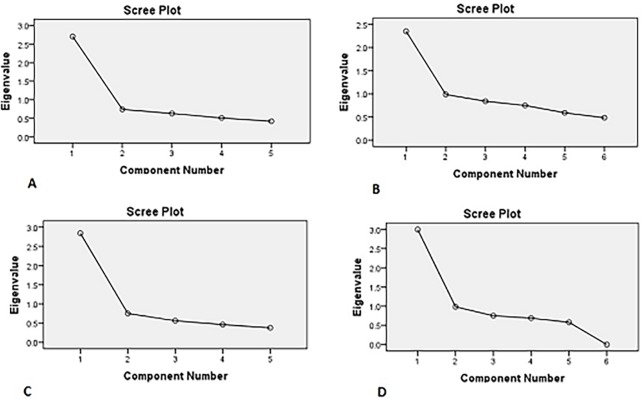
Scree plots of parent BMQ necessities (A) and concerns (B); child BMQ necessities (C) and concerns (D).

Principal component analysis was conducted for the 6 items of the parent MARS questionnaire and 5-items of children MARS questionnaire. The Kaiser-Meyer-Olkin measure verified the sampling adequacy for the analysis. Bartlett’s test of sphericity indicated that correlations between the items were sufficiently large for PCA. One component had an Eigenvalue over Kaiser’s criterion of 1 and explained (39.1%-56.8%) of the variance (Table 2). The scree plot also supported retaining one component by indicating one main point of inflection (Figs 1 and 2).

**Table 2:** Principal component analysis results for MARS and BMQ-specific for parents and children.

	MARS^a^	MARS^b^	BMQ-Necessities^a^	BMQ-Concerns^a^	BMQ- Necessities^b^	BMQ- concerns^b^
**KMO**	0.682	0.751	0.818	0.744	0.833	0.760
**% of variance**	40.5	51.9	54.1	39.1	56.8	39.6
**Bartlett’s test of sphericity**	263.2	282.3	332.4	221.4	302.1	166.4
**p-value**	0.000	0.000	0.000	0.000	0.000	0.000

KMO: The Kaiser-Meyer-Olkin.

a: Parent translation.

b: Child translation.

#### 3.2.2 Reliability and consistency of the scales

The Cronbach alpha coefficient was computed as a measure of the internal consistency of the scales and it was found to be ≥0 .7 for both BMQ-specific and MARS (Table 3).

**Table 3:** Reliability coefficient.

Scale name	N of Items	Cronbach's Alpha
**MARS-Parent**	6	0.7
**MARS-Child**	5	0.76
**BMQ-Parent**	11	0.70
**BMQ-Parent (Necessity)**	5	0.78
**BMQ-Parent (Concern)**	6	0.67
**BMQ-Child**	11	0.75
**BMQ-Child (Necessity)**	5	0.81
**BMQ-Child (Concern)**	6	0.69

Reliability of a questionnaire is commonly determined using a test-retest approach. A total of 209 parents and 198 children completed the questionnaires for the second time after 2–4 weeks from the previous clinical visit (response rates were 81.0% and 95.2% for parents and children respectively). As shown in Table 4, scores were reliable over the tested time interval. High test–retest reliability was found for MARS items and BMQ-specific items at time 1 and time 2 (ICCs varying from 0.706–0.888). The Pearson product moment correlation coefficient ranged from 0.546 to 0.805 for the entire sample (Table 5). There was a significant moderate to strong positive correlation between MARS items, BMQ-specific scales at time 1 and time 2. This indicates that the child and parent Arabic translations of the MARS and BMQ-specific are reliable.

**Table 4:** Test–retest reliability as assessed by ICC for MARS, BMQ-specific Items for parents and children.

Items	N	Intraclass correlation coefficient (95% CI)
**MARS-parent**	209	0.888 (0.852–0.915)
**MARS-child**	198	0.785 (0.715–0.838)
**BMQ-Necissty**^a^	209	0.731 (0.646–0.795)
**BMQ-Concern**^a^	209	0.739 (0.657–0.801)
**BMQ-Necissety**^b^	196	0.749 (0.667–0.811)
**BMQ-Concern**^b^	196	0.706 (0.610–0.778)

a: Parent translation.

b: Child translation.

**Table 5:** Pearson Product Moment Correlation between MARS, BMQ-specific at Time 1 and Time 2.

Items	N	Pearson correlation (r)	Sig (2-tailed
**Total MARS-parent**	209	0.805(**)	**0.000**
**Total MARS-child**	198	0.653(**)	**0.000**
**BMQ- Necessity**^a^	209	0.573(**)	**0.000**
**BMQ-Concern**^a^	209	0.589(**)	**0.000**
**BMQ- Necessity**^b^	196	0.604(**)	**0.000**
**BMQ-Concern**^b^	196	0.546 (**)	**0.000**

** Correlation is significant at the 0.01 level (2-tailed).

a: Parent translation.

b: Child translation.

### 3.3 Adherence (MARS) and beliefs (BMQ-specific) about medication of parents and children

**Fig 3:**
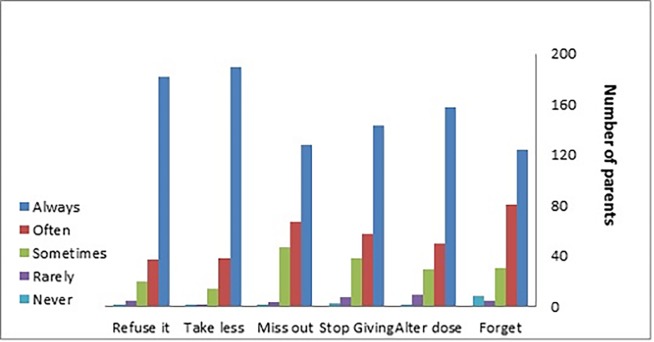
Parent reported adherence of their child to their medication [n = 249].

**Fig 4:**
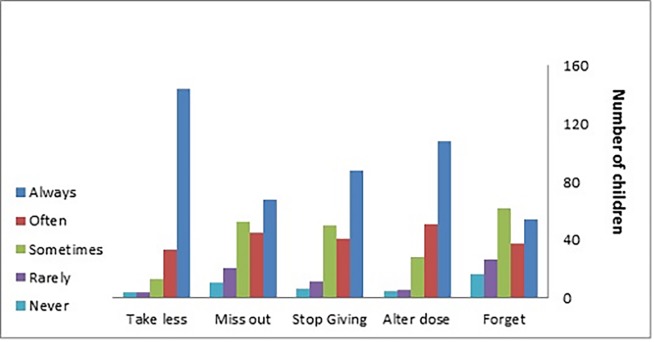
Child reported adherence to their medication [n = 198].

The MARS was completed by 249 parents and 208 children at time 1 (Figs 3 and 4). The cut point used for the MARS-parent and the MARS-child score was ≥80% (i.e. if the score was ≥ 24 for parent and score ≥20 for child this was considered adherent). The total mean MARS scores achieved were 26.49 (SD 3.3) and 20.08 (SD 3.8) for parents and children respectively. The proportion of children classified as adherent to their medication, as reported by parents using the MARS was 67.5% (n = 249); the corresponding data for MARS-children indicated an adherence level of 59.7% (n = 208).

The BMQ-Specific was completed by 258 parents and 198 children. The data were presented in Table 6, higher scores indicate stronger beliefs about the necessity or concern regarding the treatment. Parents had moderate beliefs regarding the necessity of their children's prescribed medicines however, they had somewhat stronger concerns about potential adverse effects of prescribed medication [mean 2.2 (SD 0.8); mean 2.8 (SD 0.6) respectively]. This pattern of beliefs were similar among their children who were more concerned about the harmful effect of medicines prescribed (mean 2.5, SD 0.6) compared to their beliefs in the necessity of the medicines prescribed for their specific condition (mean 2.1, SD 0.8).

**Table 6:** Beliefs about medication descriptive statistics.

	N	Median	IQR*	Range
**Parent BMQ Specific-Necessity**	258	2.2	1.6–2.8	(1–4.8)
**Parent BMQ Specific-Concern**	258	2.8	2.3–3.2	(1–4.5)
**Child BMQ Specific-Necessity**	198	2.2	1.6–2.8	(1–4.0)
**Child BMQ Specific-Concern**	198	2.5	2.2–3.0	(1–4.0)

*IQR: interquartile range.

Children who were deemed adherent had higher necessity scores (mean 2.3, SD 0.7) compared to non-adherent children (mean 1.9, SD 0.7; p<001). Also, parents whose children were classified as adherent to medication had higher necessity scores (mean 2.5, SD 0.8) compared to whose children were classified as non-adherent (mean 2.1, SD 0.8; p<001). On the other hand, no significant difference was found between adherent and non-adherent children with regard to parent/child concerns about medication (p = 0.96 for BMQ-Concerns for parents, and p = 0.17 for BMQ-Concerns for children).

## 4. Discussion

The main objectives of the present research were to produce a linguistically equivalent and culturally acceptable child and parent Arabic translation of the MARS and BMQ-specific questionnaires and assess the reliability of both questionnaires in two groups of paediatric patients (self-complete and parental completed. Additional we assessed the discriminant validity of the Arabic translation of the BMQ-Specific on the basis of whether the BMQ Specific Necessity and BMQ Specific Concerns scale scores were able to discriminate between high vs low adherence categories classified by the MARS questionnaires on a group of children with chronic diseases and their parents.

Questionnaire validation is complex and comprises multiple methods in order to find whether a certain questionnaire measures what it is intended to measure in other words: “validity refers to the appropriateness, meaningfulness, and usefulness of a measure for a specific purpose” [29].

This is the first study to assess the discriminant validity and reliability of Arabic translations of MARS and BMQ-specific. A previous study had, however, validated an Arabic translation of the BMQ-general and indicated that it has satisfactory psychometric properties in terms of its validity and reliability for measuring patient beliefs about medication [23].

The Arabic translations of the MARS questionnaire for parents and children indicated good construct validity as principal component analysis revealed that they clearly measured one construct i.e. non-adherence. This was illustrated by the distinctive scree plot and only having one component with an Eigenvalue over 1 and the fact that this component accounted for 40.5% and 51.9% of the variance in MARS-parent and MARS-child questionnaires respectively.

The validity results for the BMQ-concerns and necessity scales for parents and children were also good. All questionnaires established sampling adequacy, with all KMO values being ≥0.68. The scree plots clearly showed that one key component was measured.

Reliability of questionnaires is a measure of the consistency of results obtained. In the current study, both the ICC and the Pearson's correlation coefficient were used to quantitate reliability, however, the ICC test is usually considered superior to Pearson's r as it reflects both random and systematic differences in test scores [30]. High test–retest reliability were found (ICC>0.706). Pearson product moment correlation coefficient ranged from 0.546 to 0.805 which indicates a significant moderate to strong positive correlation between MARS items, BMQ items at time 1 and time 2. According to Greoge and Mallery (2003), a scale has an acceptable to good internal consistency, with Cronbach alpha coefficients between 0.7 ≤ α < 0.9, a criterion that was achieved in the majority of the subscales [31]. Overall, the results of this study showed that the Arabic translations of the MARS and the BMQ-Specific questionnaires are consistent and reliable.

A lot of attention has been given recently to the concept of "patient empowerment" in healthcare. Utilising this approach, patients should have an important role in making informed choices in any issue that affects their health. In terms of patient empowerment in management of their pharmacotherapy, patients should be considered as active decision makers. As such, individuals are more likely to take medication (or give medication in the case of parents of younger children) as instructed by their health care provider if they believe that its necessity outweighs their concerns about potential adverse events [32, 33]. This actually is the basic principle of the necessity–concern framework (NCF) [32]. A large number of researchers have confirmed this framework in various disease domains such as cystic fibrosis, hypertension and asthma [16, 34–36]. On the other hand, other researchers have shown a positive correlation between self-reported adherence and necessities but not concerns [37, 38].

In the current research, parents and children had higher scores for concerns compared to necessity; nevertheless adherence was correlated with necessity beliefs and was independent of the concerns regarding medications. Of the factors that could have accounted for the lack correlation between concerns about medication and adherence in the present study is the fact that a heterogeneous group of patients with various chronic diseases that could have a wide spectrum of severity participated in the research. Unfortunately, due to practical reasons, disease severity was not assessed in the present study. Another reason could be that participants may have also been reluctant to fully express their concerns about medications or participants report non-adherence, as they may have viewed this as a lack in trust in their health care provider [16].

The importance of the present study stems from it being the first to assess the validity and reliability of Arabic translations of the MARS and BMQ-specific questionnaires. The questionnaires were translated to standard Arabic i.e. no specific Arabic dialect was used, which will allow these questionnaires to be utilised not only in Jordan but in all Arabic speaking countries. Further multicentre, multinational studies are planned to assure generalisability of the current results.

Measurement of the degree of adherence and creating innovative plans for tackling the problem of non-adherence is of paramount importance in clinical care of patients with chronic diseases. Such plans are likely to be more successful in achieving their goals if they take into account beliefs about medications. In the case of children, this criterion pertains to both children themselves and the parents/guardians who are responsible for encouraging adherence or in the case of young children, administering the medication.

## 5. Limitations

Confirmatory factor analysis for MARS and BMQ-specific was not conducted to see latent factors that exert causal effect on the observed variables.

## 6. Conclusion

The Arabic translations of both BMQ-specific and MARS proved to be consistent, valid and reliable tools that could be used by researchers in clinical practice to measure adherence and beliefs about medications in Arabic speaking patient populations. The latter would help clinicians to provide individualised tailored adherence support to patients.
